# Diffusion Kurtosis Imaging Fiber Tractography of Major White Matter Tracts in Neurosurgery

**DOI:** 10.3390/brainsci11030381

**Published:** 2021-03-17

**Authors:** Miriam H. A. Bopp, Julia Emde, Barbara Carl, Christopher Nimsky, Benjamin Saß

**Affiliations:** 1 Department of Neurosurgery, University of Marburg, Baldingerstrasse, 35043 Marburg, Germany; julia.arhelger@web.de (J.E.); Barbara.Carl@helios-gesundheit.de (B.C.); nimsky@med.uni-marburg.de (C.N.); sassb@med.uni-marburg.de (B.S.); 2 Center for Mind, Brain and Behavior (CMBB), 35043 Marburg, Germany; 3 Department of Neurosurgery, Helios Dr. Horst Schmidt Kliniken, Ludwig-Erhard-Strasse 100, 65199 Wiesbaden, Germany

**Keywords:** diffusion tensor imaging, diffusion kurtosis imaging, fiber tractography, corticospinal tract, optic radiation, arcuate fascicle

## Abstract

Diffusion tensor imaging (DTI)-based fiber tractography is routinely used in clinical applications to visualize major white matter tracts, such as the corticospinal tract (CST), optic radiation (OR), and arcuate fascicle (AF). Nevertheless, DTI is limited due to its capability of resolving intra-voxel multi-fiber populations. Sophisticated models often require long acquisition times not applicable in clinical practice. Diffusion kurtosis imaging (DKI), as an extension of DTI, combines sophisticated modeling of the diffusion process with short acquisition times but has rarely been investigated in fiber tractography. In this study, DTI- and DKI-based fiber tractography of the CST, OR, and AF was investigated in healthy volunteers and glioma patients. For the CST, significantly larger tract volumes were seen in DKI-based fiber tractography. Similar results were obtained for the OR, except for the right OR in patients. In the case of the AF, results of both models were comparable with DTI-based fiber tractography showing even significantly larger tract volumes in patients. In the case of the CST and OR, DKI-based fiber tractography contributes to advanced visualization under clinical time constraints, whereas for the AF, other models should be considered.

## 1. Introduction

Diffusion tensor imaging (DTI) and DTI-based fiber tractography have become routine tools for estimating and visualizing the course, location, and extent of major white matter tracts, such as the corticospinal tract (CST), optic radiation (OR), and arcuate fascicle (AF), especially in neurosurgical applications. So far, the application of DTI and DTI-based fiber tractography has been shown to support the concept of maximized tumor volume resection, whilst preserving neurological functions, thereby contributing to low postoperative morbidity [1,2,3,4]. DTI-based fiber tractography allows well-estimated fiber bundle directions using common fiber tracking techniques and is still the most widely applied tractography method in the neurosurgical setting. However, there are fundamental limitations in accurately outlining major white matter tracts incorporating multi-fiber populations, and underestimating their spatial extent [5,6], thereby affecting the surgical decision on how to safely maximize the extent of resection.

There are several artifacts and pitfalls in diffusion weighted imaging (DWI)-based fiber tractography that one has to be aware of when applying and interpreting tractography results. These issues arise at different stages within the processing pipeline, ranging from data acquisition to visualization. The most commonly used imaging technique, echo planar imaging, itself has various artifacts such as head bulk motion and eddy currents [7] or susceptibility artifacts [8,9,10], physiological motion [11,12], partial volume effects due to typical low spatial resolution [13], or a low signal-to-noise ratio [14]. Besides the application of various fiber tractography algorithms and visualization techniques, the most crucial part, however, remains the mathematical description of the diffusion properties within each voxel. Routinely, and especially in the clinical context, a second-order tensor model (DTI) is used under the assumption of Gaussian distribution of water molecules, thus regularly failing in resolving crossing/kissing/fanning fibers [15,16] as well as complex fiber architectures due to the estimation of only a single diffusion direction within each voxel. Further complex and sophisticated methods of modeling diffusion properties exist, based on single- or multi-shell high-angular resolution diffusion imaging (HARDI) acquisition techniques [17,18], applying different models such as Q-ball imaging [19], diffusion spectrum imaging (DSI) [20], or compressed sensing techniques [21,22,23]. In most cases, these further complex techniques coincide with longer acquisition times, which makes them unsuitable for routine clinical applications [18].

Even though there are various modern and sophisticated methods available in basic neuroscience for reconstructing major white matter tracts in neurosurgical procedures, there is somehow a delay of integration of these methods, which might be due to complexity and time-consuming data acquisition or processing pipelines.

Diffusion kurtosis imaging (DKI), as an extension of DTI, enables the estimation of the diffusion tensor and the kurtosis tensor to characterize additional non-Gaussian diffusion properties within complex biological tissues such as white matter [24,25,26]. Kurtosis describes the peakedness of the probability distribution in comparison to the Gaussian distribution [27]. The estimation of the diffusion orientation distribution function (dODF) using both tensors, diffusion and kurtosis, is one alternative approach to resolve crossing fibers and to overcome the specific limitation of DTI-based approaches [26,28,29,30]. DKI typically makes use of an additional high b-value of about 2000 s/mm^2^, thereby always enabling an estimation of the diffusion tensor and associated metrics such as fractional anisotropy (FA). Given this prerequisite, DKI might be further useful for clinical applications considering scanning time and derived parameters of interest.

Even though it is clinically applicable, so far, DKI has not been widely investigated in neurosurgical applications. In recent neuro-oncological and neurosurgical applications, DKI has mostly been evaluated for its capability of glioma grading and differentiation of gliomas from other intra-axial brain tumors [31,32,33,34], its role in molecular profiling such as its correlation with IDH1/2 mutations, ATRX or Ki-67 expression [33,35], its potential as a biomarker [36], or its capability of detecting microstructural changes related to white matter alterations [37,38,39]. One study used DKI for survival prediction in glioma patients by investigating the mean kurtosis as the relevant parameter [40].

Up to now, DKI-based fiber tractography of major white matter tracts, particularly in neurosurgical applications, has been rarely investigated. In a study by Glenn et al., data of five healthy volunteers were evaluated with respect to different acquisition schemes focusing on crossing fibers [41]. Another study by Leote et al. analyzed DKI data in a group of nine patients, showing more voluminous fiber tractography results of the CST using DKI and demonstrating the feasibility of CST visualization in healthy volunteers and a small group (n = 3) of astrocytoma patients [42,43]. Another study investigated three healthy volunteers for fiber tractography along the internal capsule and corpus callosum, showing improved fiber-crossing resolution [44].

As there is still a lack of integration of sophisticated fiber tractography approaches in neurosurgical practice, DKI itself seems to be a clinically applicable and easy-to-use method, while also gaining information for standard DTI analysis. The rare previous studies only investigated initial results on tractography of the CST in glioma patients as the most widely examined white matter tract. Other neurosurgically relevant major white matter tracts such as the arcuate fascicle, associated with language processing, and optic radiation, of special interest in temporal lobe and epilepsy surgery, with several challenges in tractography algorithms were not included in those studies. Up to now, there is no gold standard available providing guidance on a suitable acquisition, model, reconstruction algorithm, and visualization that should be used for different kinds of white matter tracts.

In this study, the effect of DKI- vs. DTI-based fiber tractography of three neurosurgically relevant major white matter tracts, with different demands on the tract reconstruction, the CST, OR, and the AF, was analyzed. Analyses were performed in healthy volunteers as well as in glioma patients, specifically challenging tractography due to altered physiology (infiltration, edema), with respect to tract volume and visualization capabilities in order to evaluate DKI’s potential to support the application of fiber tractography of neurosurgically relevant white matter tracts under clinical time constraints in contrast to DTI.

## 2. Materials and Methods

### 2.1. Healthy Volunteers

20 healthy subjects (mean age: 24.50 ± 2.06 years, male/female ratio: 10/10) were included in this retrospective study to evaluate the impact of DKI-based fiber tractography in relation to DTI-based tractography. Written informed consent was obtained from all subjects after complete description of the study procedures (experimental setting, mechanism, and risks of magnetic resonance imaging (MRI) data acquisition). The study protocol was approved by the local ethics committee of the University of Marburg according to the Declaration of Helsinki (reference no. 09/13).

### 2.2. Patients

Routine clinical data of 16 patients (mean age: 55.67 ± 11.44 years, male/female ratio: 14/2) with radiological diagnosis of glioma were used retrospectively to evaluate the impact of DKI-based fiber tractography in relation to DTI-based tractography in pathological cases with varying tumor locations: left temporal: 8; left temporo-parietal: 1; left frontal: 1; left parietal: 1; left multilocular: 1; right temporal: 2; right trigonal: 1; and right parieto-occipital: 1. Pathologies included (recurrent) glioblastoma World Health Organization (WHO) IV (n = 8), anaplastic astrocytoma WHO III (n = 5), anaplastic oligodendroglioma WHO III (n = 2), and diffuse astrocytoma WHO II (n = 1).

### 2.3. MRI Data Acquisition

All MRI data sets (healthy volunteer and patient data) were acquired using a 3T MRI system (Tim Trio, Siemens, Erlangen, Germany) equipped with a 12-channel head matrix Rx-coil. Data acquisition included a T1-weighted magnetization-prepared rapid gradient echo (MPRAGE) sequence as well as a single DWI data set using a single-shot echo planar imaging sequence with the following parameters:

1. T1-MPRAGE: repetition time (TR), 1900 ms; echo time (TE), 2.26 ms; inversion time (TI), 900 ms; field of view (FoV), 256 mm; matrix, 256 × 256; slice thickness (ST), 1 mm; flip angle, 9°; 176 slices; and parallel imaging (GRAPPA) with factor 2
2. DWI: TR, 8500 ms; TE, 101 ms; FoV, 256 mm; matrix, 128 × 128; ST, 2 mm; distance factor, 0%; 60 slices; GRAPPA with factor 2; 30 diffusion encoding gradients; high b-values, 1000 and 2000 s/mm^2^; axial slices; phase encoding direction anterior >> posterior; resulting voxel size, 2 × 2 × 2 mm^3^

Data acquisition took about 14 min per subject. The DWI image volume was aligned in parallel to the connecting line of the anterior and posterior commissures within the sagittal view, as well as in parallel to the midsagittal plane, covering the entire cerebrum. All data sets were visually inspected. In case visual inspection led to at least one volume with severe artifacts, the subject was excluded from the study cohort. However, in this study, one volunteer needed to be excluded due to artifacts within the data, resulting in a new study population of 19 healthy volunteers (mean age: 24.52 ± 2.12 years, male/female ratio: 10/9).

The subjects were positioned in the supine position, head first, in the MRI scanner in a dimmed environment. Each subject’s head was positioned in prolongation of the body-line and fixated with soft foam rubber pads in order to minimize head movements as much as possible and to standardize the head position across subjects. The nasal bone was centered at the isocenter of the magnetic field.

### 2.4. Data Preprocessing

**Figure A1:**
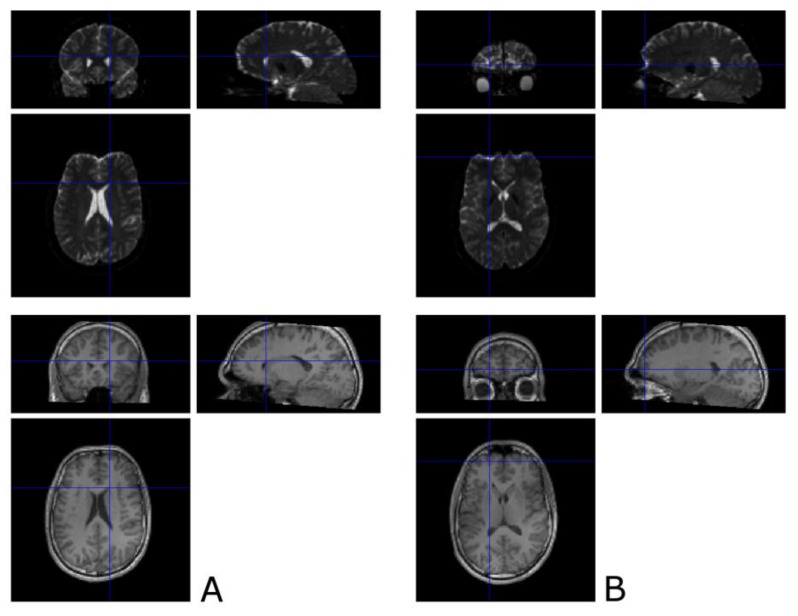
Visual inspection of registration quality (DWI: diffusion weighted imaging, T1) using the CheckReg tool implemented in Statistical Parametric Mapping (SPM) comparing the location of several anatomical landmarks such as the edge of the ventricles (**A**), showing a good match, and the frontal border (**B**) within the DWI data, showing a slight mismatch in the T1-weighted data sets due to non-linear distortions within the DWI data. This mismatch will be compensated for by the definition and enlargement of regions of interest for fiber tractography.

First, all acquired data sets were corrected for head bulk motion and eddy currents using the Eddy Current and Motion Correction (ECMOCO) toolbox [7] applying affine transformations with standard parameters. To preserve orientation information after realignment, the b-matrix was reoriented accordingly [45]. In addition, the DWI data sets were co-registered onto the individual T1-weighted image set using Co-Registration implemented in Statistical Parametric Mapping (SPM) 12 (http://www.fil.ion.ucl.ac.uk/spm/, accessed on 31 August 2020) applying a rigid transformation based on normalized mutual information. The registration quality was visually inspected (J.E.) using the CheckReg tool implemented in SPM, comparing the location of some clear anatomical landmarks such as the edges of ventricles in the registered data sets (see Figure A1). Registration inaccuracies due to non-linear distortions within the DWI data, possibly affecting fiber tractography results, were compensated for, as described later in Section 2.7.

### 2.5. Diffusion Tensor and Kurtosis Estimation

After correction for head bulk motion and eddy currents, the realigned data including the updated b-matrices were processed using Diffusion Kurtosis Estimator (DKE) software version 2.6 (Center for Biomedical Imaging, Medical University of South Carolina, https://www.nitrc.org/projects/dke/, accessed on 31 August 2020), implementing the methods of [46] applying standard parameters (spatial smoothing and strong median filtering). DTI and DKI tensor fitting were performed following the provided standard protocol (DTI: linear weighted fitting, DKI: constrained linear weighted fitting), resulting in DTI- and DKI-based parameter maps as well as tensor and kurtosis estimates.

### 2.6. Whole-Brain Fiber Tractography

Using diffusion and kurtosis tensors, whole-brain fiber tractography was performed using the DKE Fiber Tractography Module (Center for Biomedical Imaging, Medical University of South Carolina, https://www.nitrc.org/projects/dke/, accessed on 31 August 2020), implementing the methods provided by [29,41]. Orientation distribution function optimization was performed using standard parameters, as provided in the software manual. For tractography, besides recommended parameters, the thresholds were set to 0.05 for fractional anisotropy, 40° for tract angles, and 50 mm for the minimum tract length for DTI- and DKI-based tractography being performed in parallel. Results of fiber tractography were then visualized using TrackVis 0.6 (R. Wang, J. van Wedeen, Martinos Center for Biomedical Imaging, Massachusetts General Hospital, http://trackvis.org/, accessed on 31 August 2020) [47].

### 2.7. Seed Regions and Selection of White Matter Tracts

To outline the CST, OR, and AF, appropriate regions of interest (ROIs) were defined. For the CST, typically reported ROIs are the motor cortex (MC) as well as the cerebral peduncle (CP) [48,49]. For tractography of the OR, ROIs covering the lateral geniculate nucleus (LGN) as well as the visual cortex (Brodman areas 17–19) have been frequently reported in the recent literature on ROI-based selection of the OR [50,51,52]. For tractography of the AF, one standardized approach is to investigate color-coded FA maps to outline a region covering the horizontal part of the AF lateral to the CST within coronal FA images and a region covering the descending portion of the AF in the posterior temporal lobe [53,54].

**Figure A2:**
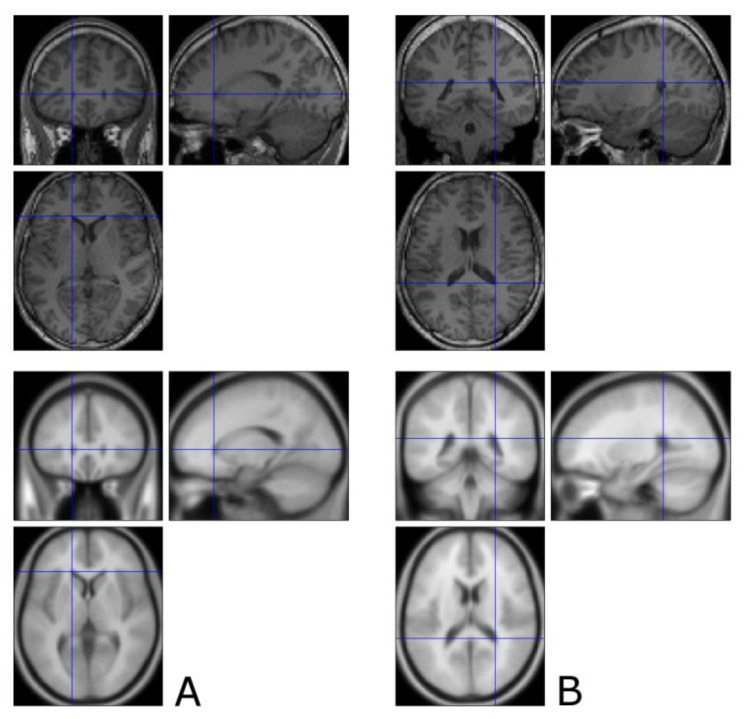
Visual inspection of registration quality during normalization using the CheckReg tool implemented in SPM comparing the location of several anatomical landmarks such as the edges of the ventricles ((**A**): ventral part, (**B**): dorsal part), showing a good correspondence between the template space (lower part) and the non-linearly deformed single-subject T1-weighted image (upper part).

For the AF and the cerebral peduncle, ROIs were defined manually within the corresponding regions. For ROIs covering the motor cortex, visual cortex, and LGN, an atlas-based approach was used applying the Jülich histological (cyto- and myelo-architectonic) atlas [55,56,57] implemented in the FMRIB Software Library (FSL) (Oxford, United Kingdom) [58,59,60], mapped to the Montreal Neurological Institute (MNI) template space. To transform these ROIs to individual volunteer and patient data, anatomical data (T1) of each subject was spatially normalized into the MNI template space using SPM 12. The spatial normalization thereby encompasses a linear transformation (12-parameter affine registration), leading to a rough alignment of the data, followed by non-linear transformation (warping), deforming the anatomical data to account for smaller-scale differences in both data sets. The registration accuracy was again visually inspected (J.E.) using CheckReg, as described before (see Figure A2). The inverse transformation was then used to transform the ROIs within the MNI template space to the individual single-subject image space.

**Figure A3:**
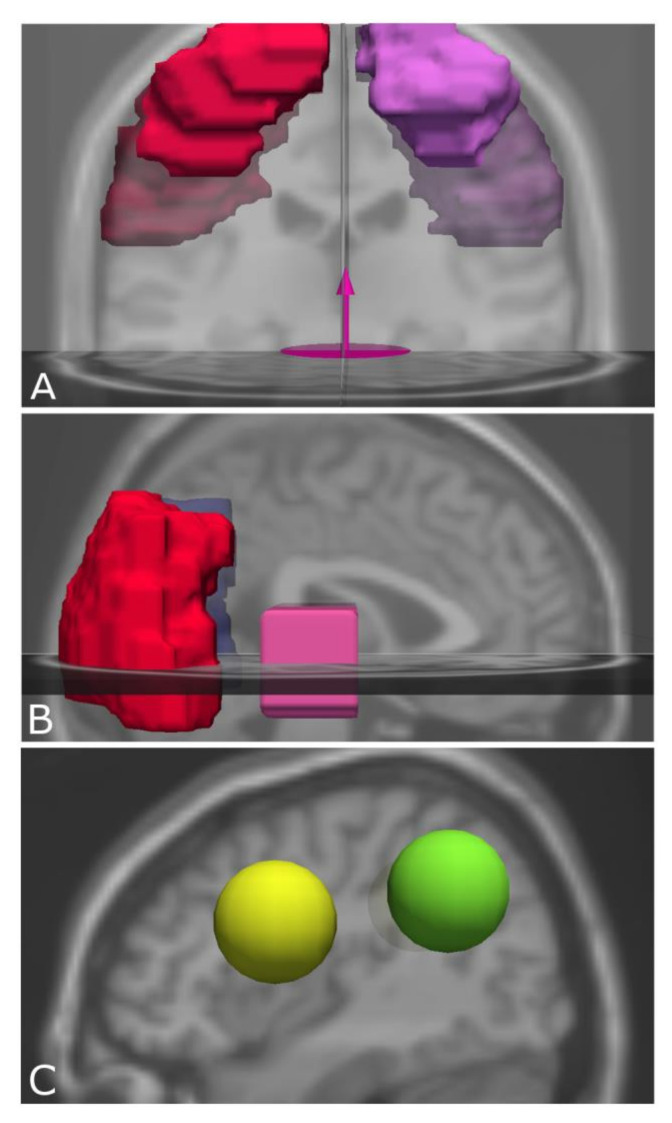
Definition of regions of interest (ROIs) for the CST (**A**), covering the precentral gyrus as well as the cerebral peduncles; for the OR (**B**), covering the visual cortex and the lateral geniculate nucleus (LGN); and for the AF (**C**), covering the regions of the horizontal part and the descending portion of the AF.

To account for registration inaccuracies between T1-weighted data and DWI data due to especially non-linear distortions, as well as between T1-weighted data and the template, all ROIs were enlarged. In the case of atlas-based ROIs covering the motor cortex, visual cortex, and LGN, the ROIs were enlarged by 10 mm in the x, y, and z directions. In the case of ROIs used for tractography of the AF, 40 mm 3D spheres were used and centered according to the FA map. For the cerebral peduncle (bilateral), a 40 mm 2D circular ROI was used covering the cerebral peduncles in an axial slice (see Figure A3).

**Figure A4:**
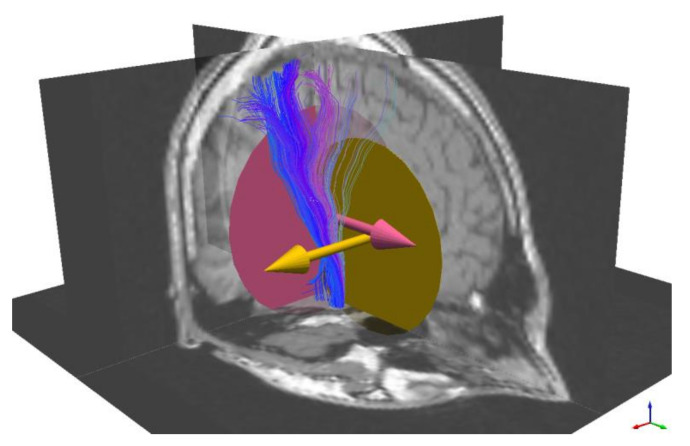
Definition of exemplary two exclude regions (2D circles) for initial refinement of the CST tractography results to eliminate fibers connecting to the cerebellum (pink ROI) and fibers crossing to the other hemisphere (yellow ROI).

For reconstruction of the CST, OR, and AF based on DTI and DKI whole-brain tractography, the corresponding ROIs were used as include regions. After initial fiber tractography based on the two include regions for each tract, additional ROIs (exemplary see Figure A4) were manually defined and applied as exclude regions (the same for DTI and DKI) using 2D circles of different sizes in the axial, coronal, or sagittal view to remove fibers not being part of the tract of interest according to recent neuroanatomical knowledge gained from dissection and tractography studies (e.g., postmortem studies) [61,62,63,64,65,66]. Besides single fibers that were removed individually for all tracts using this approach, in the case of the CST, mainly fibers visualized within the cerebellum, post-motor cortex, and supplementary motor cortex and fibers connecting to the other hemisphere needed to be excluded. In the case of the OR, some fibers in the first place reconstructed based on the two seed ROIs connecting the frontal and occipital lobes (e.g., part of the inferior fronto-occipital fascicle) were excluded, as well as fibers medial to the ventricles, not being part of the OR. In the case of the AF, fibers running medial, in parallel to the AF, needed to be excluded, as well as fibers connecting to the anterior frontal lobe. As it is performed by a single user, this way, the user-dependent fiber tract refinement is standardized.

Finally, fiber tract volumes [16] were calculated in order to compare the results of DTI- and DKI-based fiber tractography, and all tracts were visually inspected. To further quantify visual inspection, fiber density (number of fibers divided by number of voxels in the cross-sectional area of the tract) was calculated for the CST at the level of the internal capsule (axial cross-section), for the OR at the level of the posterior boundary of the corpus callosum (coronal cross section), and for the AF in a coronal slice in the middle between both seed regions (coronal cross section). In the case of the CST, the motor cortex was subdivided into a medial, a medial-to-lateral and a lateral component, whereas the second and third component are most often not reached properly by DTI-based approaches. To quantify fanning of the tracts, cases reaching only the medial component, the medial and medial-to-lateral components, or the medial, medial-to-lateral, and lateral components were counted.

### 2.8. Statistical Analysis

Statistical analyses were performed using SPSS Statistics version 26 (IBM, Armonk, NY, USA) using the Shapiro–Wilk test to test for normal distribution of differences between groups as a prerequisite for the paired *t*-test. In the case of no normal distribution, the Wilcoxon signed-rank test was applied and α was set to 0.05. To correct for multiple comparisons for the left vs. right side, the significance level was set to α/2 (*p* < 0.025).

## 3. Results

### 3.1. The Corticospinal Tract

For healthy volunteers as well as patients, significantly larger tract volumes were seen in DKI-based tractography compared to DTI-based tractography of the left and right CST. Detailed information about the mean and standard deviation (SD) as well as the minimum and maximum (range) is given in Table 1 and Table 2.

**Figure 1:**
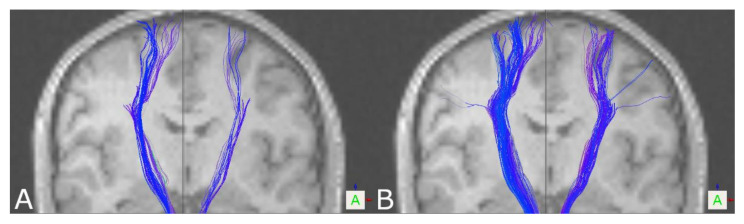
Tractography results of the CST for a healthy volunteer based on DTI (**A**) and DKI (**B**), showing a broader fanning within the motor cortex, also sparsely connecting to the lateral part of the motor cortex (coronal view).

Visually inspecting the tractography results of the CST in healthy volunteers and patients, overall reconstructions based on DKI seemed to provide more solid and compact tract reconstructions compared to reconstructions based on DTI (see Figure 1). Tract density calculated at the level of the internal capsule (number of fibers divided by cross-sectional area of the tract (fibers/voxel)) underpinned this impression, showing significant differences between DTI- and DKI-based tractography for the left and right CST in healthy volunteers as well as in patients. For further details on fiber tract density, see Table 1 and Table 2.

**Figure 2:**
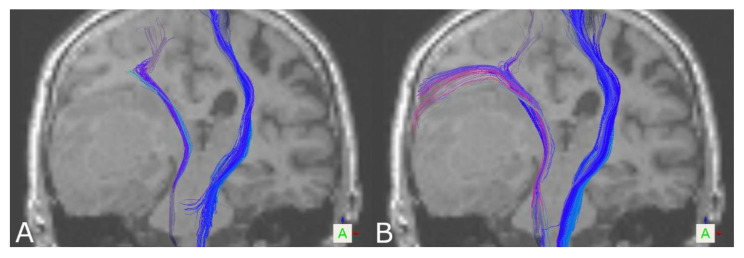
Tractography results of the CST based on DTI (**A**) and DKI (**B**) for a patient with a right temporal glioblastoma WHO IV, in close vicinity of the CST (coronal view).

Additionally, visual inspection showed a broader fanning of tracts within the motor cortex, partially also connecting to the lateral part of the motor cortex based on DKI. Thereby, for the left CST in 16/19 healthy volunteers and 11/16 patients, DKI-based tract reconstructions reached a broader part of the motor cortex, including the medial-to-lateral component; for the right CST, this was the case in 15/19 healthy volunteers and 12/16 patients. Connections to the lateral part were seen based on DKI in four healthy volunteers/five patients for the left CST and three healthy volunteers/three patients for the right CST (see Figure 1). In four patients, the left or right CST was in close vicinity of a lesion or affected by peritumoral edema, altering diffusion characteristics (see Figure 2). In all four cases, DKI-based reconstructions led to further neuroanatomically plausible tractography results, whereas in these pathological cases, DTI partially almost failed to visualize tracts at all.

### 3.2. The Optic Radiation

In healthy volunteers, significantly larger tract volumes were seen in DKI-based fiber tractography rather than DTI-based fiber tractography (see Table 3). In patients, significantly larger tract volumes based on DKI were only seen for the left but not the right OR (see Table 4).

**Figure 3:**
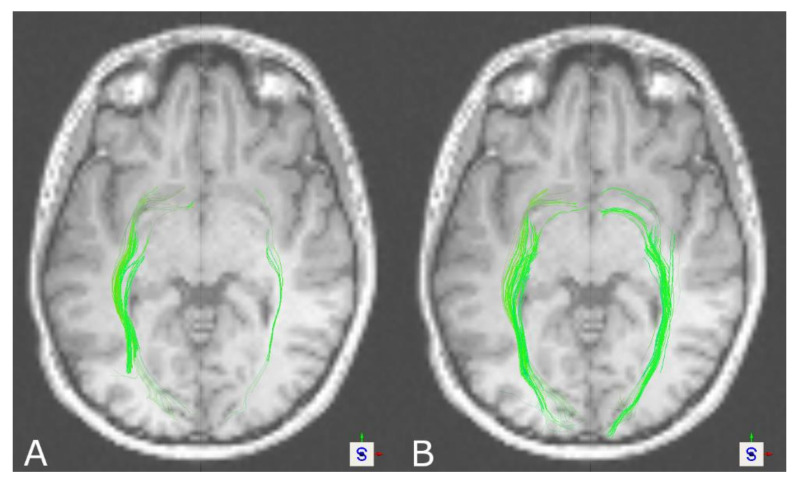
Tractography results of the OR for a healthy volunteer based on DTI (**A**) and DKI (**B**), showing more solid and compound tract reconstructions in the case of DKI (axial view).

Visually inspecting tractography results of the OR, DKI-based tractography overall seemed to show more solid and compact reconstructions compared to DTI-based tractography for the left and right OR in healthy volunteers (see Figure 3). Tract density calculated at the posterior boundary of the corpus callosum in a coronal slice (number of fibers divided by cross-sectional area of the tract) underpinned this impression, showing significant differences between DTI- and DKI-based tractography for the left and right OR in the case of healthy volunteers, whereas in patients, no significant difference was seen either in the left or in the right OR (see Table 3 and Table 4).

In 7 of 16 patients, the left or right OR was in close vicinity of a lesion or affected by perilesional edema, and in 5 of those cases, DKI showed further anatomical plausible tractography results compared to the DTI-based approach.

### 3.3. The Arcuate Fascicle

In the case of healthy volunteers, DTI- and DKI-based fiber tractography of the AF revealed no significant differences in tract volume (see Table 5). For patients, significantly larger tract volumes were seen in DTI-based fiber tractography compared to DKI-based fiber tractography of the AF (see Table 6).

**Figure 4:**
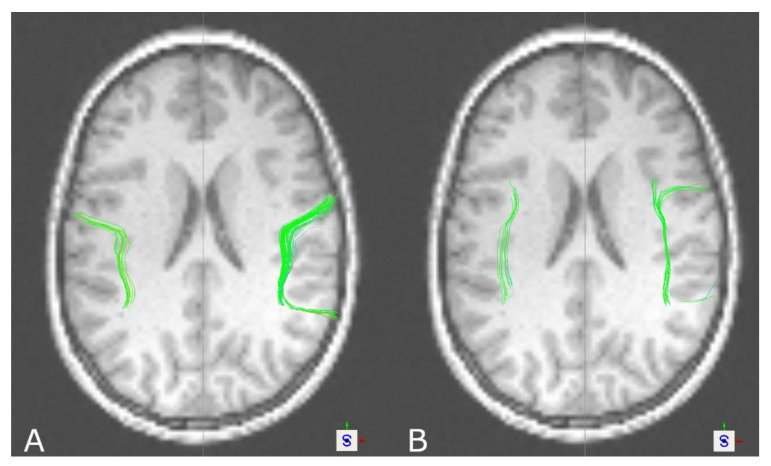
Tractography results of the AF for a healthy volunteer based on DTI (**A**) and DKI (**B**), showing more solid and compound tract reconstructions based on DTI (axial view).

Visual inspection of AF reconstructions showed neuroanatomically further plausible fiber tract reconstructions in favor of DTI-based tractography rather than DKI-based tractography. Especially in the case of patients with a significant difference in tract volume, but also in single cases in healthy volunteers where no significant overall difference in tract volume was found, slightly more compact tract reconstructions were seen based on DTI (see Figure 4). Tract density was calculated in a coronal slice centered between the two seed regions (number of fibers divided by cross-sectional area of the tract) but overall underpinned this impression only in the case of patients for the left AF, showing significant differences between DTI- and DKI-based tractography, whereas for the right AF, no significant difference was seen. In the case of healthy volunteers, no significant differences in tract density were seen for the left or the right AF. For further details, see Table 5 and Table 6.

Furthermore, visual inspection showed that DTI-based tractography was rather capable of visualizing the partially curved tracts connecting both ROIs, which was seen for the left AF in 15/19 healthy volunteers and 11/16 patients and for the right AF in 12/19 healthy volunteers and 12/16 patients. In patients, the left AF was affected by a lesion or perilesional edema in three cases and the right AF also in three cases.

## 4. Discussion

Tractography of the CST led to significantly larger tract volumes based on DKI in healthy volunteers and patients. On visual inspection of the results, reconstructions seemed more solid and compact based on DKI, which was also seen regarding tract density. Additionally, DKI-based reconstructions in contrast to DTI-based reconstructions were frequently capable of reaching a broader part of the motor cortex, in some cases connecting also to the lateral part of the motor cortex. In addition, in the case of the OR in healthy volunteers, significantly larger tract volumes were seen in DKI-based tractography. Similarly, this was seen in patients for the left, but not the right, OR. In the case of the AF, the results seem to be the opposite. Larger tract volumes, even though not significant in healthy volunteers, were seen in favor of DTI-based tractography, also going along with further neuroanatomically plausible reconstructions. Due to a lack of ground truth data to compare against for verification of the results, besides the tract volume as one of the most commonly used measures of comparing tractography results between groups or algorithms to interpret the results, tract reconstructions were assessed visually, underpinned by a measure of tract density at a representative localization.

There is a general consensus for further sophisticated methods to be applied for reconstruction and visualization of major white matter tracts in order to overcome the limitations of the routinely used DTI model [5,67,68]. Several approaches exist to define which model and method to use in which scenario, and various approaches have been compared [69,70,71,72], notably showing, so far, the weakest results for DTI-based methods. Most methods therefore rely on extensive acquisition times (e.g., single-/multi-shell HARDI data) to generate complex data sets [72,73] not suitable for clinical use. DKI only extends DTI using an additional high b-value, approximately doubling the required acquisition time. Even though HARDI acquisition is also possible for DKI, the use of 30 directions is recommended to estimate sufficient DKI metrics at shorter acquisition times [41,74]. However, DKI is usually not included in these comparative studies, even though DKI extends the DTI model using kurtosis to model non-Gaussian diffusion properties.

Whereas most studies focus on DKI’s capability of detecting pathological alterations more precisely than by using DTI, studies including DKI-based tractography are, so far, rare. Leote et al. investigated DKI-based fiber tractography based on 1.5 T MRI data, not being evaluated before, for reconstructions of the CST in eight healthy volunteers as well as three glioma patients [42]. No difference between tract sizes of patients and healthy volunteers was seen, but no comparison of DTI- and DKI-based tractography regarding tract sizes was performed. In another study, Leote et al. compared DTI- and DKI-based fiber tractography of the CST in nine patients with space-occupying lesions [43]. Overall, more voluminous fiber tracts were seen based on DKI, as well as curvilinear fibers above the lesion not visible based on DTI in five cases, underpinning the findings of the present study. Another study compared tractography and analyzed the number of streamlines within the corpus callosum and internal capsule and showed improved fiber-crossing resolution in all three healthy subjects in the case of the corpus callosum but not of the internal capsule [44]. Glenn et al. compared DTI, DKI, and DSI data in three healthy volunteers [75], with a major focus on the methods’ capability of resolving complex fiber microarchitecture rather than tractography results. They showed that DKI enables the detection of crossing fibers, resulting in pronounced improvements compared to DTI. Results also indicated comparable results across DKI and DSI data, suggesting DKI can be used in a clinical setting due to reduced acquisition times. Results of DKI-based tractography regarding tract volumes of the CST are in line with the previous literature, whereas DKI-based tractography on the AF and OR is not reported so far.

Fiber tractography of the OR is notoriously challenging due to its neuroanatomical complexity, low fiber density, sharp curvature, high variability, and fan-like structure [76,77,78]. In addition, the temporal stem contains multiple fibers impacting accurate delineation from each other [79,80,81], and some technical aspects such as low spatial resolution, low signal-to-noise ratio, and susceptibility artifacts might compromise tractography of the OR [78]. As previously reported, DTI-based reconstructions of the OR often suffer from inaccuracies, with several levels of success of tractography [82,83]. Neto Henriques et al. showed the potential of DKI to resolve fiber crossings of variable angulation in contrast to DTI. Therefore, complex fiber architectures such as the OR should also be resolved more adequately using DKI [30]. This seems to be in concordance with the findings of this study, where at least more solid and compact reconstructions were seen using DKI compared to further sparse representations given by the DTI-based approach, which was also seen in significantly larger tract volumes and tract density in healthy volunteers (left and right OR). However, in patients, analogous results regarding tract volume were only seen for the left but not the right hemisphere. This might be caused by the study cohort that included cases with tumors especially affecting this tract and thereby possibly lowering the effect. Brain tumors cause histological distortion and increase microstructure complexity [24]. Leote et al. postulated in the case of the CST that especially in regions affected by a brain tumor, DTI metrics are more affected than DKI metrics, leading to the hypothesis that DKI-based tractography is more suitable to characterize the underlying microstructure [42].

Surprisingly, in the case of the AF, larger tract volumes were seen in favor of DTI-based tractography yielding no significant differences in healthy volunteers but in patients. In DKI-based fiber tractography, reconstructions often stopped early and frequently did not connect the ROIs properly. With a lack of other studies on DKI-based tractography of the AF, results can be explained only speculatively. Neto Henriques et al. analyzed the 3D geometry of the diffusion kurtosis tensor and proposed that tractography results might depend on the orientation of the fiber bundle and its curvature. Ascending fibers seem to follow more concave and convex pathways and should reflect small fanning angles to be better resolved by DKI-based approaches [30], which might not be the case for the AF, with its close relation to other language-related tracts, such as the superior longitudinal fascicle. Lazar et al. hypothesized that with the increasing number of fiber directions, especially almost perpendicular crossings can be resolved better than crossings with small angles [26]. In the case of the AF with other tracts running nearby with similar fiber directions, visualizing those tracts might be affected. In this way, DKI-based tractography might not be suitable for the AF. This might also be in line with results reported by Loucao et al., who compared DTI- and DKI-based tractography in different ROIs and showed opposed results in favor of DTI or DKI [44], or results reported by Neto Henriques et al., who compared the kurtosis tensor vs. DKI-based tractography, leading to opposed results in different regions (corpus callosum, internal capsule) [30]. This also supports the above-mentioned idea on defining which model and method to use in which scenario in order to find the best-fitting approach for every patient [69,70,71,72].

Even though DKI-based tractography of the CST and OR seems to possibly overcome some drawbacks of the DTI-based approach, tract appearance (visual inspection regarding fanning/curvature, tract density) varied across the cases, e.g., not all cases showing fanning within the motor cortex in healthy volunteers and patients. Besides tract-specific demands on tractography algorithms, there might also be subject-specific challenges in the tractography approach, such as decreased/altered white matter integrity related to pathological conditions (tumor, edema) or aging (reduced FA [84]), as patients in this retrospective study were significantly older than the cohort of heathy volunteers. In addition, post-processing-related issues could be considered, such as artifacts and registration inaccuracies. In the case of the AF, contrary to the CST and OR, DKI seems to be even less capable of resolving the AF, while the DTI-based approach in these cases leads to plausible results. This even further supports the idea of individualized approaches depending on the specific subject’s and tract’s demands and incorporating shortcomings of image processing steps. Even though not for all tracts, DKI seems to be one possible approach to overcoming some drawbacks of DTI under clinical time constraints, worthy to be further investigated for specific white matter tracts, such as the CST and OR, while other approaches need to be investigated for other tracts such as the AF.

However, there is a need for approaches considering not only physiological challenges but also clinical constraints (compliance, short acquisition times). Considering various sophisticated approaches already available in basic neuroscience, there is still a delay of integration in neurosurgical procedures, mainly due to time-consuming data acquisition and complex processing pipelines. DKI, however, still provides short acquisition times, easily being integrated in the clinical workflow, and working as an extension of DTI, it still provides an estimation of the diffusion tensor and DTI-associated metrics used in routine clinical practice. Supported by the results of this study, especially in case of the CST but also the OR, DKI seems to support the neuroanatomically plausible visualization of fiber tract resection, even when DTI only leads to sparse reconstruction or even fails to provide plausible reconstructions at all, and could thereby be a valuable complement to DTI-based tractography.

So far, there is no real general gold standard for acquisition, parameter definition, or post-processing pipelines (post-processing, model, tractography algorithm, visualization, etc.) for the analysis of DWI data and fiber tractography. In this way, for example, even scanner manufacturer, field strength, and MR acquisition parameters influence DTI-derived parameters that are used for fiber tractography, such as scan repetitions, choice of the maximum b-value, number of diffusion-encoding gradients, the gradient-sampling scheme, image resolution, and echo and repetition times [85], and have to be kept in mind when analyzing and interpreting study results. Another important general issue arises from the use of the b-matrix, usually automatically generated by the scanning system. These typically do not consider, e.g., imaging gradient effects and coupling of imaging and diffusion gradients and provide constant b-matrix values across the whole imaging volume, leading to systematic errors, as previously described by Borkowski et al. [86] and Krzyzak et al. [87]. In those studies, a new approach called b-matrix spatial distribution in DTI (BSD-DTI) was presented investigating an anisotropic phantom with ground truth knowledge of spatial distribution of the diffusion tensor to gain information about the voxel-by-voxel spatial dependence of the b-matrix, independent of sequence-specific parameters. Comparing the application of the traditional scanner-related b-matrix and the BSD-DTI-based b-matrix, systematic intra- and interslice deviations are seen, leading to a more accurate determination of diffusion coefficients as well as the orientation of the diffusion tensor, thereby also affecting DTI-based parameters and related fiber tractography results. Even though only applied in some recent studies [88,89], applying a BSD-DTI phantom-based approach to accurately defining the b-matrix and therefore also to more accurately estimating the diffusion tensor characteristics and related information could, in general, be considered to reduce uncertainty within the data.

However, certain limitations of the study should be mentioned that should be addressed in further investigations to support and broadly quantify these findings. First, limitations could be found in DWI data themselves, such as low spatial resolution, reduced signal-to-noise ratio, and artifacts due to susceptibility or pulsation, that might be accounted for to reduce physiological and physical bias. For example, a higher TE due to an increased maximum b-value, as used here, in comparison to acquisition of a standard DTI data set (maximum b-value of 1000 s/mm^2^) might lead to a lower signal-to-noise ratio and increased susceptibility and distortion artifacts and thereby also might affect scalar parameters such as the FA, typically used for fiber tractography [85,90,91]. Non-linear geometric distortions, for example, could be addressed as they are supposed to affect the tractography of all three tracts, which can be accounted for by acquisition of additional DWI data with an inverse phase encoding direction. Additionally, the patient cohort was heterogeneous due to the location of the space-occupying lesion, with tracts variably being affected by a tumor or not. In addition, in this retrospective study, age significantly varied between healthy volunteers and patients, affecting diffusion parameters, even though no direct comparison was performed. Finally, there was a lack of a true gold standard to verify and validate fiber tractography, as tract volume, tract density, and tract appearance, not yielding immediate quantitative information on anatomical correctness. To validate the results, phantom studies (simulation or hardware model) or ex vivo specimens (e.g., postmortem MRI and dissection) can be used [92], but nevertheless, the application and evaluation in vivo needs to be further investigated, for example, using intrasurgical mapping techniques in a larger cohort of patients.

## 5. Conclusions

In this study, DKI- and DTI-based fiber tractography of three major white matter tracts, the CST, OR, and AF, was investigated in healthy volunteers and glioma patients to evaluate the potential of DKI to overcome limitations of DTI-based fiber tractography, often underestimating the spatial extent of major white matter tracts. The resulting fiber tracts were inspected visually due to neuroanatomical plausibility and appearance, as well as objectively compared using tract volume and fiber density.

In the case of the CST, DKI-based tractography led to more solid and compound tracts with a larger tract volume, also, in part, being capable of visualizing fibers connecting to the lateral motor cortex. Even in the absence of comparable studies in the case of the OR, our results are also in line with the theory of DKI’s capability to resolve multi-fiber populations, especially of tracts crossing more or less perpendicularly, leading to enhanced tract visualization based on DKI. For the AF, inverse results were found, with DTI-based fiber tractography seeming to be comparable or even more plausible than DKI-based tractography according to visual inspection and tract volume. Particularly in patients, this effect is more considerable. As DKI-based fiber tractography of the AF has not been investigated so far, one can only speculate that DKI might be compromised in cases of multi-fiber populations crossing in small angles and in cases of strong curvature, such as for the AF.

These results (solid and compact vs. sparse reconstructions, along with larger tract volumes) indicate that DKI-based tractography possesses the potential to contribute to improved fiber tractography of the CST and OR in routine clinical practice, especially under clinical time constraints, compared to DTI but needs to be further investigated and evaluated in clinical applications (e.g., intraoperative stimulation for verification). However, for the AF, alternative approaches need to be explored to overcome the limitations of DTI-based fiber tractography, supporting the general consensus to define specific models and methods for application of single tract reconstructions to optimize white matter tract visualization. Nevertheless, DKI seems to be a promising method, complementary to DTI, to provide robust visualization of selected major white matter tracts such as the CST and OR.

## Figures and Tables

**Table 1:** Results of fiber tractography of the corticospinal tract (CST) in healthy volunteers.

	Diffusion Tensor Imaging (DTI)		Diffusion Kurtosis Imaging (DKI)		DTI vs. DKI	
	Left CST	Right CST	Left CST	Right CST	Left CST	Right CST
Tract volume (cm^3^)						
Mean ± SD	13.34 ± 5.49	14.78 ± 5.36	18.57 ± 5.82	19.86 ± 5.47	*p* < 0.001 *	*p* < 0.001 *
Range (min; max)	(3.89; 22.64)	(6.67; 25.61)	(8.70; 28.70)	(13.34; 29.88)		
Tract density (fibers/voxel)						
Mean ± SD	6.74 ± 1.90	6.63 ± 1.99	8.49 ± 2.30	8.34 ± 2.51	*p* < 0.001 *	*p* = 0.002 ^+^
Range (min; max)	(1.85; 10.67)	(3.69; 9.52)	(3.70; 13.03)	(3.94; 13.92)		

* Paired *t*-test (normal distribution of differences according to the Shapiro–Wilk test). ^+^ Wilcoxon signed-rank test (no normal distribution of differences according to the Shapiro–Wilk test).

**Table 2:** Results of fiber tractography of the CST in patients.

	DTI		DKI		DTI vs. DKI	
	Left CST	Right CST	Left CST	Right CST	Left CST	Right CST
Tract volume (cm^3^)						
Mean ± SD	11.08 ± 4.58	10.74 ± 5.10	18.50 ± 6.17	18.44 ± 6.53	*p* < 0.001 *	*p* < 0.001 *
Range (min; max)	(2.87; 17.25)	(3.53; 21.99)	(4.04; 26.27)	(6.62; 34.17)		
Tract density (fibers/voxel)						
Mean ± SD	3.99 ± 1.57	4.34 ± 1.64	7.81 ± 2.58	8.23 ± 2.40	*p* < 0.001 *	*p* < 0.001 *
Range (min; max)	(1.62; 6.70)	(1.25; 7.57)	(3.71; 11.88)	(4.59; 11.84)		

* Paired *t*-test (normal distribution of differences according to the Shapiro–Wilk test).

**Table 3:** Results of fiber tractography of the optic radiation (OR) in healthy volunteers.

	DTI		DKI		DTI vs. DKI	
	Left OR	Right OR	Left OR	Right OR	Left OR	Right OR
Tract volume (cm^3^)						
Mean ± SD	5.74 ± 3.10	3.69 ± 2.40	7.64 ± 2.78	5.02 ± 2.33	*p* < 0.001 *	*p* = 0.005 *
Range (min; max)	(1.11; 12.02)	(1.10; 10.78)	(3.28 to 14.18)	(0.74 to 10.40)		
Tract density (fibers/voxel)						
Mean ± SD	2.84 ± 1.16	2.31 ± 0.80	3.48 ± 1.11	2.98 ± 1.19	*p* = 0.005 ^+^	*p* = 0.02499 *
Range (min; max)	(1.00; 5.68)	(1.00; 4.26)	(1.09; 5.90)	(1.00; 5.64)		

* Paired *t*-test (normal distribution of differences according to the Shapiro–Wilk test). ^+^ Wilcoxon signed-Rank Test (no normal distribution of differences according to the Shapiro–Wilk test).

**Table 4:** Results of fiber tractography of the OR in patients.

	DTI		DKI		DTI vs. DKI	
	Left OR	Right OR	Left OR	Right OR	Left OR	Right OR
Tract volume (cm^3^)						
Mean ± SD	4.07 ± 3.16	4.45 ± 3.23	5.43 ± 3.81	4.81 ± 2.61	*p* = 0.021 *	*p* = 0.389 *
Range (min; max)	(0.38; 12.29)	(0.99; 14.10)	(0.35; 16.62)	(2.54; 13.42)		
Tract density (fibers/voxel)						
Mean ± SD	2.77 ± 1.43	2.17 ± 1.18	2.91 ± 1.13	2.57 ± 1.12	*p* = 0.638 *	*p* = 0.183 *
Range (min; max)	(1.00; 5.46)	(0.25; 4.00)	(1.25; 4.63)	(1.12; 4.73)		

* Paired *t*-test (normal distribution of differences according to the Shapiro–Wilk test).

**Table 5:** Results of fiber tractography of the arcuate fascicle (AF) in healthy volunteers.

	DTI		DKI		DTI vs. DKI	
	Left AF	Right AF	Left AF	Right AF	Left AF	Right AF
Tract volume (cm^3^)						
Mean ± SD	4.04 ± 1.74	3.31 ± 1.23	3.21 ± 1.73	2.61 ± 1.26	*p* = 0.101 *	*p* = 0.044 ^+^
Range (min; max)	(1.50; 7.38)	(1.12; 5.46)	(0.80; 6.43)	(0.42; 5.18)		
Tract density (fibers/voxel)						
Mean ± SD	4.90 ± 2.35	4.56 ± 2.68	3.84 ± 2.05	3.61 ± 1.85	*p* = 0.163 *	*p* = 0.205 *
Range (min; max)	(2.25; 10.56)	(0.29; 11.43)	(1.14; 9.67)	(1.05; 6.71)		

* Paired *t*-test (normal distribution of differences according to the Shapiro–Wilk test). ^+^ Wilcoxon signed-rank test (no normal distribution of differences according to the Shapiro–Wilk test).

**Table 6:** Results of fiber tractography of the AF in patients.

	DTI		DKI		DTI vs. DKI	
	Left AF	Right AF	Left AF	Right AF	Left AF	Right AF
Tract volume (cm^3^)						
Mean ± SD	5.02 ± 2.66	4.64 ± 1.97	3.66 ± 1.60	2.97 ± 1.09	*p* = 0.023 *	*p* = 0.010 *
Range (min; max)	(0.58; 8.98)	(2.40; 8.75)	(0.00; 5.55)	(1.58; 5.45)		
Tract density (fibers/voxel)						
Mean ± SD	6.53 ± 4.37	4.81 ± 1.91	3.57 ± 1.84	4.65 ± 2.72	*p* = 0.010 *	*p* = 0.823 *
Range (min; max)	(0.60; 15.09)	(1.08; 7.78)	(0.00; 6.55)	(1.06; 9.70)		

* Paired *t*-test (normal distribution of differences according to the Shapiro–Wilk test).

## Data Availability

The data presented in this study are available on reasonable request from the corresponding author. The data are not publicly available due to ethical reasons.
